# Arginine Metabolism and Its Potential in Treatment of Colorectal Cancer

**DOI:** 10.3389/fcell.2021.658861

**Published:** 2021-05-20

**Authors:** Tao Du, Junyi Han

**Affiliations:** Department of Colorectal Surgery, East Hospital, Tongji University School of Medicine, Pudong, China

**Keywords:** arginine metabolism, transporters protein, signal pathway, colorectal cancer, stem cells

## Abstract

Colorectal cancer is the leading cause of death from cancer globally. The current treatment protocol still heavily relies on early detection and surgery. The molecular mechanisms underlying development of colorectal cancer are clinically important and determine the prognosis and treatment response. The arginine metabolism pathway is hyperactive in colorectal cancer and several molecules involved in the pathway are potential targets for chemoprevention and targeted colorectal cancer therapy. Endothelial nitric oxide synthase (eNOS), argininosuccinate synthetase and ornithine decarboxylase (ODC) are the main enzymes for arginine metabolism. Limiting arginine-rich meat consumption and inhibiting ODC activity largely reduces polyamine synthesis and the incidence of colorectal cancer. Arginine transporter CAT-1 and Human member 14 of the solute carrier family 6 (SLC6A14) are overexpressed in colorectal cancer cells and contributes to intracellular arginine levels. Human member 9 of the solute carrier family 38 (SLC38A9) serves as a component of the lysosomal arginine-sensing machinery. Pharmaceutical inhibition of single enzyme or arginine transporter is hard to meet requirement of restoring of abnormal arginine metabolic network. Apart from application in early screening for colorectal cancer, microRNA-based therapeutic strategy that simultaneously manipulating multiple targets involved in arginine metabolism brings promising future in the treatment of colorectal cancer.

## Introduction

Colorectal cancer is a highly prevalent and highly fatal disease worldwide, with a third incidence rate and fourth mortality rate (Brenner et al., 2014). While with decreasing trends for the incidence and mortality of colorectal cancer in many developed countries like the United States, the incidence and mortality in several developing countries, such as China, have continued to increase (Center et al., 2009a,b). These trends have been ascribed to their transition toward a so-called western lifestyle such as the consumption of high-fat diets and physical inactivity (Center et al., 2009b).

The molecular pathogenesis of colorectal cancer is heterogeneous including hereditary components and developing slowly through adenoma–carcinoma sequence in most cases. The molecular mechanisms underlying development of colorectal cancer are clinically important because they are associated with the prognosis and treatment response of the patient (De Sousa et al., 2013; Sadanandam et al., 2013). At present, the treatment of colorectal cancer is mainly surgery based comprehensive treatment, but the treatment effect of recurrent or metastatic colorectal cancer is still very limited (Cunningham et al., 2010; Pritchard and Grady, 2011). Recently, genomics and proteomics have made a lot of progress in the diagnosis and treatment of colorectal cancer (Manna et al., 2014), especially in the study of polyamines and their metabolism related molecules (Gerner and Meyskens, 2004; Manna et al., 2014).

It is well known that arginine is an original source of polyamines generation, and abnormal arginine metabolism has been characteristic of tumor cell metabolism. As a semi-essential amino acid in humans, arginine is critical for the growth of human cancers, particularly in those with chemoresistance and poor clinical outcomes. In addition to protein synthesis, arginine is involved in diverse aspects of tumor metabolism, including the synthesis of nitric oxide, polyamines, nucleotides, proline and glutamate. In addition, several enzymes and transport molecules in the arginine metabolic pathway such as ODC, CAT, and SLC6A14 were involved in the development of tumors, including colorectal cancer. There have also been some recent reviews on arginine metabolism in tumors, such as epigenetic arginine regulation in antitumor therapy and arginine deprivation. These reviews analyzed the role of arginine metabolism in tumors from various perspectives. However, studies have shown the two-faced effect of arginine. Some studies confirm that arginine enhances tumor growth (Selvi et al., 2019), others introduce it as an appropriate candidate for cancer treatment (Jahani et al., 2017). Currently, there is a lack of systematic review of the role of arginine metabolism in colorectal cancer. Besides, the effect of arginine metabolism in CRC is uncertain. In recent years, several studies on arginine metabolism in colorectal cancer have been emerged successively. For example, it has been found that limiting arginine-rich meat intake and inhibiting ODC activity can reduce polyamine synthesis and colorectal cancer incidence, and endothelial nitric oxide synthase (eNOS) inhibitors can inhibit CRC cell proliferation. In addition, miRNAs were an important class of molecules involved in multiple steps of tumor development (Niu et al., 2018). Thus, miRNAs targeting arginine and metabolic-related enzymes would be used as potential diagnostic markers or therapeutic targets. In order to better understand arginine metabolism and its role in diagnosis and therapy for colorectal cancer, this review discusses arginine metabolism pathway involved enzymes and its transporters in colorectal cancer. Although the literature on arginine metabolism in colorectal cancer was limited, we hope that this review will provide guidance for the diagnosis and treatment of clinical colorectal cancer, such as finding specific markers for the diagnosis and managing arginine intake in patients with high-risk factors for colorectal cancer.

## The Role of Arginine and Its Metabolites in CRC

Arginine is a semi essential amino acid for human body. The arginine is generated by two ways under physiological conditions, one is the ornithine cycle and the other is the membrane protein transport receptor to transfer extracellular arginine to the cell (Gerner and Meyskens, 2004). Many enzymes and arginine transporters were involved in the metabolism of arginine. Arginine could generate ornithine through Arg-1 and ornithine was involved in polyamine synthesis. Arginine could also generate guanidine through arginine decarboxylase (ADC) and then participate in cell signal pathway. Guanine can be produced under the action of arginine deaminase (ADI) or nitric oxide synthetase. Besides, arginine could be synthesized again through the arginine succinate synthetase (ASS1) and arginine succinate lyase (ASL) (Szlosarek, 2014). Once the metabolism of arginine is broken, it is easy to cause tumor (Battaglia et al., 2014; Paz et al., 2014).

Arginine and Its metabolites play an important role in the development of CRC. Studies have found that limiting arginine-rich foods could reduce the incidence of colon cancer. Recently, it has been reported that that CRC cell lines could not grow in arginine free medium *in vitro*, and DNA replication stopping and cyclin expression down-regulation were also identified, which could be reversed by exogenous arginine supplementation (Alexandrou et al., 2018). In addition, expression of ASS was significantly increased in CRC, while overexpression of ASL was negatively correlated with prognosis (Huang et al., 2015, 2017).

Arginine is the substrate of eNOS. It was found that eNOS in CRC was related to tumor vascular invasion (Chhatwal et al., 1994), and eNOS inhibitors could inhibit the proliferation and apoptosis of CRC cells through downstream molecules (Altun et al., 2013). ODC could decompose the metabolites of arginine into polyamines, which was necessary for the development and proliferation of CRC (Gerner and Meyskens, 2009). The expression of ODC in CRC was significantly increased, which could regulate the cell cycle process and promote tumor progression (Nakanishi et al., 1993). Meanwhile, ODC inhibitors could reduce the occurrence of colon polyps and adenomas (Battaglia et al., 2014). In addition, arginine transporters were also involved in the development of CRC. SLC6A14 expression was up-regulated in CRC which involved in the regulation of mTOR signaling pathway, thereby regulating cell proliferation and energy metabolism (Gupta et al., 2005). Inhibition of CAT-1 could reduce the survival rate of tumor cells and inhibit the expression of EREG, which was a key factor in the transformation from inflammation to colon cancer (Camps et al., 2013). Furthermore, some drugs that induce the expression of SAT1 spermidine/spermidine N1 acetyltransferase (SAT1 or SSAT) had therapeutic effects on inflammatory CRC, indicating that they might also be involved in the regulation of the progress of CRC (Evageliou and Hogarty, 2009; Goodwin et al., 2011).

## Intracellular Arginine Metabolic Pathway in CRC and Its Implication for Therapies

**FIGURE 1:**
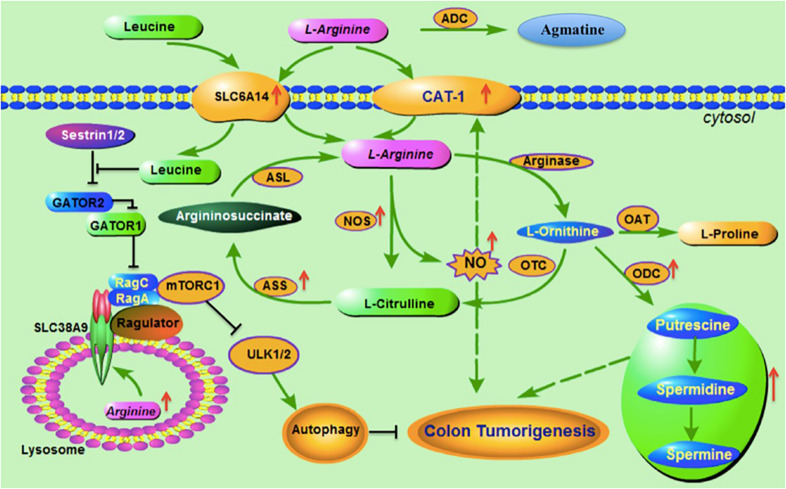
Arginine metabolic pathways and association with colorectal cancer. Before L-arginine enters into cells, arginine decarboxylase (ADC) catalyzes the arginine to generate agmatine. Arginine transporters SLC7A1 (CAT-1) and SLC6A14 are the main transporters responsible for arginine transmembrane transportation. Both CAT-1 and SLC6A14 are highly expressed in colorectal cancer. SLC6A14 is also responsible for transportation of leucine. Nitric oxide synthase (NOS) catalyzes the cytosol L-arginine to generate L-citrulline and Nitric oxide (NO), and the latter is reported to upregulate CAT-1 expression. L-citrulline is converted into argininosuccinate via Argininosuccinate synthetase (ASS), which is a rate-limiting enzyme in arginine recycle biosynthesis. Argininosuccinate lyase (ASL) catalyzes argininosuccinate to produce endogenic L-arginine. Arginase catalyzes the L-arginine to generate L-ornithine, which has three metabolic pathays: (1) converting to L- citrulline via Ornithine transcarbamylase (OTC); (2) to generate L-proline via Ornithine aminotransferase (OAT); and (3) to produce polyamines (including putrescine, spermidine and spermine) via ornithine decarboxylase (ODC). NOS, polyamines and ODC are all reported upregulated in colorectal cancer and contributing to colon tumorigenesis. SLC38A9 is a component of the lysosomal arginine sensing machinery and sestrin1/2 is the sensor of cytosol leucine, both of which control mTORC1 and regulate autophagy and involve colon tumorigenesis.

L-arginine has been a long known substrate of eNOS or nitric oxide synthase 3 (NOS3), with resulting metabolic products of L-arginine–NO being nitric oxide (NO) and Citrulline (Figure 1). Recent studies demonstrated that cancer cells have a higher eNOS expression, as eNOS is required for maintaining permanent tumor growth via Ras-activated PI3K–Akt signaling pathway (Fukumura et al., 2006; Lim et al., 2008). Clinical studies on human colon cancer samples suggest that high eNOS expression can be positively correlated with tumor cell vascular invasion (Chhatwal et al., 1994), as well as in trophoblast cancer (Ariel et al., 1998). Besides, an eNOS inhibitor L-NIO could increase the antiproliferative, antiangiogenic and apoptotic effects of E7080, a tyrosine kinase inhibitor, on CRC cell *in vitro*. In was found that blocking the eNOS phosphorylation could inhibits tumorigenesis, while overexpression of eNOS enhanced the nitrosylation and activation of Ras proteins *in vitro* and vivo (Lim et al., 2008).

Arginine as is a substrate of eNOS is thus crucial for the tumor-drivinng PI3K–Akt–eNOS (wild-type)-Ras pathway, which further explains the increased arginine catabolism in cancer cells. Cellular recycling mechanisms are in place to provide sufficient substrate (citrulline) for arginine synthesis with help of intracellular argininosuccinate synthetase (ASS) and argininosuccinate lyase (ASL) (Figure 1). Loss of ASS in several tumor entities renders them arginine auxotrophic, e.g., hepatocellular carcinoma, malignant melanoma, malignant pleural mesothelioma, prostate and renal cancer (Ensor et al., 2002). In contrast, several platinum sensitive tumors, including primary ovarian, stomach and colorectal cancer, are characterized by ASS overexpression (Delage et al., 2010). This explains the inutility of arginine deprivation in colorectal cancer therapy.

L-arginine catabolized by arginase (ARG) produces ornithine, which is further broken down by ornithine decarboxylase (ODC) to polyamines (Figure 1), such as putrescine, which is essential for CRC development and proliferation (Gerner and Meyskens, 2009). In earlier studies, it was found that increased polyamine expression in colorectal cancer tissues was associated with increased ODC activity, and the ODC protein and mRNA expression were significantly higher in CRC tissue compared to paired normal tissues (LaMuraglia et al., 1986). It has been proved that ODC was engaged in G1/S progression, and the cell cycle modification by agmatine through ODC inhibition was considered indirect while by interfering with cyclins expressions, agmatine exerted a direct effect (Nakanishi et al., 1993). Furthermore, its dose-dependent inhibitory effect has been demonstrated on some cancers including colon cancer and hepatocellular carcinoma (Patil et al., 2016). Thus, it is comprehensible why an overexpression of ODC has been proven in CRC (Goodwin et al., 2011) and neuroblastoma (Evageliou and Hogarty, 2009; Battaglia et al., 2014), confirmed that ODC might promote the colorectal cancer progress (Patil et al., 2016).

Studies have found that adenomatous polyps (APC) tumor suppressor gene and KRAS gene play important roles in the process of polyamine production and colorectal tumorigenesis. Increased ODC transcription and polyamine synthesis were detected in APC mutant mice. At the same time, the use of ODC inhibitors can significantly reduce the incidence of colon polyps and adenoma (Battaglia et al., 2014), and can also be used for the chemoprevention of prostate adenocarcinoma and skin cancer (Manni et al., 2004; Xu et al., 2008). There is evidence that limiting the meat consumption and inhibiting ODC activity can significantly reduce polyamine synthesis and incidence rate of colorectal cancer with ODC1 GA/AA genotype, compared to GG (Zell et al., 2012).

## Polyamine Metabolic Pathways and Their Potential Therapeutic Targeting in CRC

Polyamine is a small molecular weight organic polycation, which can combine with negatively charged substances such as RNA, miRNA and protein, and participate in the transcriptional regulation of gene expression (Gerner and Meyskens, 2004; Battaglia et al., 2014; Paz et al., 2014). In addition, polyamines can modify the eukaryotic translation initiation factor-5A (eIF5A) and affect the transcription and translation of downstream oncogenes and tumor suppressor oncogenes (Scuoppo et al., 2012; Paz et al., 2014).

The metabolism of intracellular polyamines is strict regulated in cells. When these balances are broken, it is easy to lead to tumorigenesis such as in colorectal cancer (Battaglia et al., 2014; Paz et al., 2014; Figure 1). Spermidine/spermidine catabolism is regulated by three major enzymes, including spermidine oxidase (SMO), spermidine/spermidine N1 acetyltransferase (SAT1 or SSAT) and N1 acetylpolyamine oxidase (APAO). The drug sulindac and other NSAIDs can induce SAT1 expression in human cell and mouse models, which may be one of the reasons for the treatment effect of inflammatory colorectal cancer (Evageliou and Hogarty, 2009; Goodwin et al., 2011). Recently, it has been found that overexpression of SAT1 can rapidly reduce the levels of spermidine and spermine in cells, thereby inhibiting cell protein synthesis and preventing cell growth (Mandal et al., 2013). These results indicate that SAT1 has a certain prospect in the treatment of colorectal cancer. Similarly, a variety of catabolic enzymes involved in polyamine catabolism, including SSAT, APAO, and SMO, are also potential targets for the treatment of colorectal cancer.

## Arginine Transporters in Colorectal Cancer

Arginine can shuttle across the cell membrane through a variety of transporters (Lu et al., 2013, Figure 2). The most common arginine transporter family is that of Na^+^ -independent cationic amino acid transporters (CAT), which consists of CAT-1, -2A, -2B, -3, and -4 (Malandro and Kilberg, 1996; Palacin et al., 1998). The studies are focus on cat-1 and cat-2, while the function and specificity of CAT-3 and cat-4 are not clear (Closs et al., 2006). Another arginine transporter is the sodium- and chloride-dependent transporter, which is encoded by member 9 of solute carrier family 6 (SLC6A14 gene). In colorectal cancer, CAT-1 expression was negatively correlated with pathological grade (Figure 3).

**FIGURE 2:**
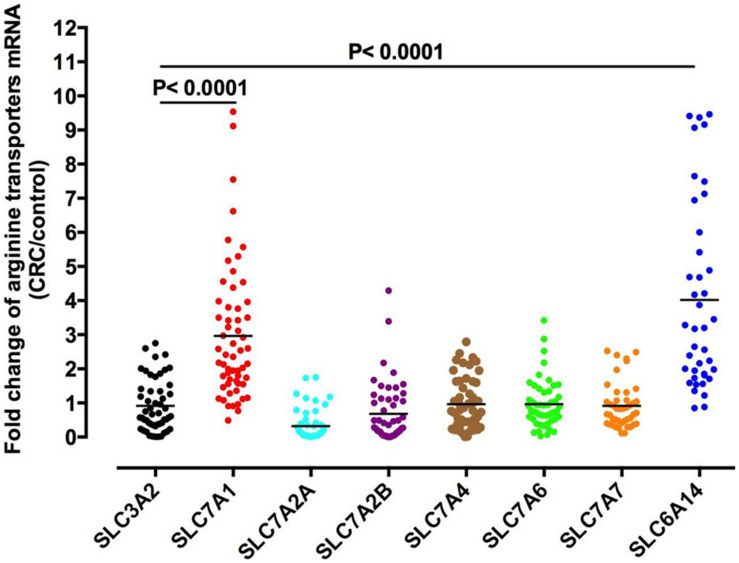
Arginine transporters expression in colorectal cancer. The mRNA levels for each arginine transporter in colorectal cancer and adjacent normal tissues of 90 colorectal cancer patients were measured by qPCR. The expression of SLC7A1 and SLC6A14 genes were significantly upregulated in colorectal cancer patients.

**FIGURE 3:**
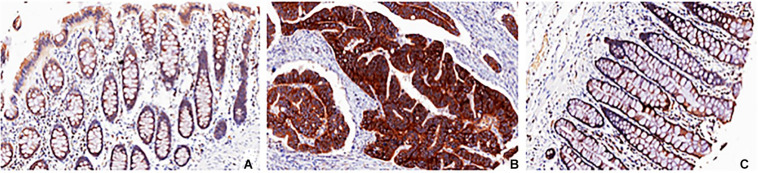
Histochemistry of SLC7A1 in colorectal cancer and control. The pathological characteristics of colon adenocarcinoma **(B)** and adjacent normal colon tissue **(C)** in the tumor specimen were shown when sectioning and staining with anti-SLC7A1 antibody and compared with normal colon tissue from control **(A)**.

Camps et al. (2013) found that siRNA down-regulation of CAT -1 expression can reduce the survival rate of cancer cells, and significantly inhibit the expression of growth factor Epiregulin (EREG), which is a key factor in the transformation of colon from inflammation to tumor. There are some differences in arginine transporters in different cells. For example, CAT -2 is mainly expressed in some immune cells such as macrophages (Morris, 2010), while CAT-1 is specifically expressed in colorectal cancer (Su et al., 2004). Since CAT -1 is a membrane protein, specific monoclonal antibodies can be used to neutralize cat-1 and inhibit the uptake of arginine by CRC cells, so as to achieve the purpose of tumor treatment.

SLC6A14 was found to be highly expressed in colorectal tissues (Gupta et al., 2005), due to the increased demand of tumor cells for leucine and arginine (Karunakaran et al., 2011). The leucine is an activator of mTOR pathway (Laplante and Sabatini, 2012; Figure 1), which can form two protein complexes mTORC1 and mTORC2, playing an important role in tumors associated with metabolic disorders. The expression and leucine transport of SLC6A14 are regulated by PKC (Closs et al., 2006), which is a downstream molecule of mTORC2 and an important signal molecule regulating tumor cell proliferation (Zell et al., 2012). Based on the above results, the inhibition of arginine uptake by SLC6A14 may have potential clinical significance. Human member 9 of the solute carrier family 38 (SLC38A9) has recently been identified as a component of the lysosomal amino acid (particular arginine)-sensing machinery that regulates the targets of rapamycin complex 1 (mTORC1) (Jung et al., 2015; Rebsamen et al., 2015; Wang et al., 2015). The mechanistic mTORC1 integrates the presence of growth factors, energy levels, glucose and amino acids to modulate metabolic status and cellular responses. Overexpression of SLC38A9 or just its Regulator-binding domain activates mTORC1 signaling even in the absence of amino acids, while loss of SLC38A9 impairs mTORC1 activation by amino acids, particularly arginine (Wang et al., 2015). It has been demonstrated that amino acids regulated mTORC1 pathway through the Rag guanine triphosphatases (GTPases), which was regulated by a positive regulator GATOR2 and its interaction protein Sestrin2. Interestingly, leucine but not arginine, disrupts the Sestrin2-GATOR2 interaction and inhibits mTORC1 signaling (Saxton et al., 2016; Wolfson et al., 2016; Figure 1). In ASS1-deficient prostate cancer cells, arginine withdrawal leads to increased protein turnover via reduced synthesis and increased breakdown (suppression of mTOR and proteosomal degradation, respectively) and triggers caspase-dependent and caspase-independent apoptotic cell death in a cell type- dependent manner (Changou et al., 2014; Szlosarek, 2014). Many cancer cell types use this autophagy-based mechanism to overcome the arginine supply problem.

## Arginine Metabolism and Stem Cells

Arginine and its metabolism related molecules are closely related to stem cells and tumor stem cells. Recent studies have shown that arginine may be closely related to the physiological function of stem cells, including cancer stem cells. L-arginine can increase the expansion of small intestinal stem cells (ISCS) by targeting rapamycin complex1 and inhibiting Wnt2B secretion in small intestinal (SI) organoid models. In addition, L-arginine therapy can protect the intestinal tract from injury (Pearce et al., 2012). Zhang et al. found that exogenous L-arginine could promote the proliferation and intestinal epithelial renewal of ISCS, and protect the gut from the injury induced by TNF-α and 5-FU in mice (Hou et al., 2020). Arginine is also involved in the differentiation of human bone marrow mesenchymal stem cells into osteoblasts and adipocytes. Arginine significantly increased the expression of osteogenic transcription factors runt related transcription factor 2 (Runx2), dix5, and osterix in MSCs, and decreased adipocyte formation and triglyceride content. This effect is associated with the increased expression of Wnt5a and nuclear factor of activated T-cells (NFATc), which could be reversed by Wnt and NFATc antagonists (Huh et al., 2014).

It is also found that eNOS is involved in the differentiation of cancer stem cells. The expression of eNOS is increased in hyperproliferative intestinal crypts, which was associated with relapse free survival and overall survival. Overexpression of eNOS decrease the proliferation and expression of tumor stem cell markers such as Lgr5 and Vav3. These data suggest that eNOS may be a potential new target in mesenchymal colorectal tumors with poor prognosis (Penarando et al., 2018). Expression and activity of eNOS change dynamically in the process of differentiation of mouse adult pluripotent progenitor cells into endothelial cells induced by vascular endothelial growth factor. The expression and activity of eNOS increase on the 14th and 21st day of differentiation (Liu et al., 2007). The NO/NOS/sGC/PKG-I pathway is also involved in the cardiac differentiation of embryonic stem cells (Spinelli et al., 2016). Further, neuronal NOS (nNOS) is involved in the differentiation of human induced pluripotent stem cells (hiPSCs). It is found that the expression of nNOS in migrating hiPSCs is down regulated by comparing the gene expression profiles of migrating and non-migrating hiPSCs, which is a related regulator of hiPSCs migration to cancer cells. Inhibiting activity of nNOS or down regulating its expression can reduce the migration of neural stem cells (NSCs) and improve their tumor tropism. This indicates that nNOS is a potential target for cancer therapy mediated by NSCs (Chen et al., 2013).

Besides arginine and NOS, arginine decarboxylase (ADC) is involved in the repair of MSCs against injury. It is found that overexpression of ADC can reduce the activation of Caspase-3, promote the phosphorylation of Akt and CREB, and increase the expression of BDNF in H2O2 treated MSCs. These results indicate that ADC can protect MSCs against H_2_O_2_ toxicity and improve the survival rate of MSCs (Seo et al., 2013). ADC is also involved in the neuronal differentiation of neural progenitor cells (NPCs). NPCs overexpressing ADC gene can differentiate by neural lineage *in vitro* model of cerebral ischemia. Transplantation of NPCs over-expressing ADC can inhibit the volume of cerebral infarction, promote neural differentiation and protrusion *in vivo*. These results suggest that ADC has potential value in cell replacement therapy of ischemic stroke (Kim et al., 2019).

## Immune Regulation of Arginine and its Metabolites in CRC

Arginine and its metabolites play an important role in the development of T cells and the maintenance of tumor microenvironment. Studies have found that arginine was very important for the formation of T cell receptor. Abnormal activation of Arg-1 could lead to the loss of arginine in the tumor environment, resulting in T cell dysplasia and the loss of tumor cell response related receptors (Rodriguez et al., 2003; Yachimovich-Cohen et al., 2010). In addition, arginine deficiency reduced the dephosphorylation level of cofilin protein and affected the recovery of actin in turn, which was necessary for the production of immune synapses and T cell proliferation (Feldmeyer et al., 2012). Furthermore, NO activated cyclooxygenase-2 (COX-2) and other inflammatory mediators, thereby creating a pro-oxidant microenvironment that supported cancer cell growth and suppressed antitumor immunity (Tham et al., 2014; Hugo et al., 2016). Besides, iNOS/NO positively regulated the production of COX-2, microsomal prostaglandin E synthase-1 (mPGES1), and prostaglandin E2 (PGE2), which was related to immune-based anticancer therapies (Feun et al., 2008).

At present, the research concerning arginine and colorectal cancer immunity was very limited. Studies have found that myeloid suppressor cells (MDSCs) in colon cancer cells could inhibit the function of Th1/Th17/Th2 lymphocytes and form an immunosuppressive environment (Kang et al., 2010; Galon et al., 2013), which was crucial for the survival of tumor cells. MDSCs could be divided into M1 and M2 types according to cell morphology and polarization state. The activity of iNOS was significantly increased in M1 type, which has anti-tumor effect (Modolell et al., 1995; Allavena and Mantovani, 2012; Fridlender and Albelda, 2012). On the contrary, the up-regulation of ARG1 level shows cancer promoting effect In M2 type (Fridlender et al., 2009; Ma et al., 2011). It was also shown that NO is required in pathogen-induced colon inflammation and immune cell infiltration, leading to dysplasia and colon cancer development (Erdman et al., 2009). In parallel, NO could activate macrophages and cytotoxic T cells, and augment the immune response against tumor cells (MacMicking et al., 1997; Marigo et al., 2016). Moreover, it has been demonstrated that macrophage-derived NO induced the expression of the adhesion molecule VCAM-1 in tumor vessels of melanoma xenografts, which is important for T-cell extravasation. Additionally, Nos2-/- macrophages could not co-transfer with CD8 + T cells yield T-cell homing to the tumor and tumor rejection (Sektioglu et al., 2016).

## miRNAs and Long Non-Coding RNAs in Arginine Metabolism and CRC

miRNAs are also involved in the regulation of arginine metabolism. miRNA can regulate arginine metabolism by regulating the expression of key molecules in arginine metabolism pathway, such as ASS (Bates et al., 2010; Tu et al., 2020), ARG1 (Bates et al., 2010; Yoo et al., 2019), ARG2 (Dunand-Sauthier et al., 2014; Jin et al., 2014; Kim et al., 2017; Wang Y. et al., 2017), CAT-1 (Chang et al., 2004; Li Y. et al., 2018), ODC (Jagannathan et al., 2015) and NOS (Perske et al., 2010; Yan et al., 2011; Guo et al., 2012; Sun et al., 2012; Zhu et al., 2013; Li et al., 2014, 2017, Li H.T. et al., 2018; Fu et al., 2015; Jiang et al., 2015; Zhang et al., 2015, 2020; Rasheed et al., 2016; Reilly et al., 2016; Muxel et al., 2017, 2018; Wang C. et al., 2017, Wang et al., 2019; Cui et al., 2020; Lin et al., 2020; Scalavino et al., 2020; Table 1). It has been found that multiple miRNAs could target the same enzyme or transporter protein, and the same molecule could also be regulated by multiple miRNAs. In addition, the regulatory mechanisms were diverse, which include binding to 3′-UTR region to degrade target genes by, inhibiting or increasing the expression of target genes or the enzyme activity at the same time. For example, microarray analysis of affinity purified RNAs and their validation identified CAT-1 as target gene of miR-122 (Bhattacharyya et al., 2006; Li et al., 2012), suggesting that arginine metabolism regulatory mechanisms are modulated by miRNA expression. Another example was the applying of ODC inhibitors to successfully reverse the LIN28/Let-7 axis and inhibit glycolytic metabolism in neuroblastoma (an entity similar to CRC in terms of arginine metabolism) (Lozier et al., 2015). Overexpression of ODC enhanced menin translation by reducing miR-29b, whereas polyamine depletion by inhibiting ODC increased miR-29b and suppressed menin expression (Ouyang et al., 2015). Since arginine metabolism is related to rectal cancer, searching the miRNAs targeted arginine metabolism—related enzyme may be new sights for the diagnosis and treatment of CRC.

**TABLE 1:** miRNAs target to arginine metabolism.

**Study**	**Target**	**miRNA identified**	**Diseases or cells**	**Methods of analysis**
Tu et al. (2020)	ASS1	miR-1291-5p	Pancreatic carcinoma	qRT-PCR
Bates et al. (2010)	ASS1	mmu-miRs-22, -127, -470, and -411	Mice	qRT-PCR
Bates et al. (2010)	ARG1	mmu-miRs-29b, -676, -382, and -669b	Mice	qRT-PCR
Yoo et al. (2019)	ARG1	miR-340-5p	Peripheral Blood Cells	Luciferase reporter assay
Wang Y. et al. (2017)	ARG2	miR-613	HCMV-positive glioblastoma	Luciferase reporter assay
Jin et al. (2014)	ARG2	miR-17-5p	Human pulmonary artery smooth muscle cell	qRT-PCR
Kim et al. (2017)	ARG2	miR-1299	Melasma	Luciferase reporter assay
Dunand-Sauthier et al. (2014)	ARG2	miR-155	T cell	Luciferase reporter assay
Li Y. et al. (2018)	CAT-1	miR-122	Isoniazid-induced liver injury	qRT-PCR
Chang et al. (2004)	CAT-1	miR-122	Primary human hepatocytes	Luciferase reporter assay
Jagannathan et al. (2015)	ODC	miR-29b	Myeloma cells	qRT-PCR
Wang C. et al. (2017)	eNOS	miR-138 and miR-199a	Rats	Luciferase reporter assay
Zhang et al. (2020)	eNOS	miR-221	Atherosclerosis	qRT-PCR
Wang et al. (2019)	eNOS	miR-155-5p and miR-24-3p	Atrial fibrillation	qRT-PCR
Li et al. (2017)	eNOS	miR-455-3p	HUVECs	qRT-PCR
Fu et al. (2015)	eNOS	miR-335 and miR-543	Prostate cancer	Luciferase reporter assay
Jiang et al. (2015)	eNOS	miR-584 and miR-335	Severe preeclampsia	Luceriferase assay
Yan et al. (2011)	eNOS	27-nt miRNA	Endothelial cell	qRT-PCR
Li et al. (2014)	eNOS	miR-155	Severe preeclampsia, HTR-8/SVneo cells	qRT-PCR
Zhang et al. (2015)	eNOS	miR-155	Human aortic SMCs (HASMCs)	Luciferase reporter assay
Sun et al. (2012)	eNOS	miR-155	Human umbilical vein endothelial cell	Luciferase reporter assay
Li H.T. et al. (2018)	eNOS	miR-24	Subarachnoid hemorrhage (SAH)	Luciferase reporter assay
Muxel et al. (2018)	iNOS	let-7e	Lamazonensis-infected	qRT-PCR
Scalavino et al. (2020)	iNOS	miR-369-3p	Inflammatory dendritic cells	qRT-PCR
Lin et al. (2020)	iNOS	miR-206-3p and miR-381-3p	Macrophages	qRT-PCR
Cui et al. (2020)	iNOS	miR-302b-5p	Parkinson’s disease	Luciferase reporter assay
Perske et al. (2010)	iNOS	miR-146a	Mouse renal cell carcinoma cell line	qRT-PCR
Guo et al. (2012)	iNOS	miR-939	Human hepatocytes	Luciferase reporter assay
Zhu et al. (2013)	iNOS	miR-26a	NPM-ALK(+) T-cell lymphoma	Luciferase reporter assay
Rasheed et al. (2016)	iNOS	miR-26a-5p	Human osteoarthritis chondrocytes	Luciferase reporter assay
Reilly et al. (2016)	nNOS	miR-31	Human atrial fibrillation	qRT-PCR
Muxel et al. (2017)	NOS2	miR-294 and miR-721	Lamazonensis-infected	qRT-PCR

Apart from argininemetabolism, specific microRNAs (miRNAs) have been identified in CRC. miRNAs are now known to be essential in malignancies, functioning as tumor suppressors and oncogenes (Kong et al., 2012). miRNAs can be used to diagnose the presence of CRC and help predict disease recurrence (Zhang et al., 2013). Differential expression of specific miRNAs sampled in tissues or plasma offers the prospect of their use in early detection and screening for colorectal cancer (Schetter et al., 2008; Ng et al., 2009; Liu et al., 2013; Luo et al., 2013; Toiyama et al., 2013; Chen et al., 2015; Table 2). miR-21, miR-92a, miR-29a, and miR-150 have strong potential as novel non-invasive biomarkers for early detection and prognosis of colorectal cancer (Huang et al., 2010; Ma et al., 2012; Wu et al., 2012). Analysis of colorectal tumors and adjacent non-neoplastic tissues from patients and colorectal cancer cell lines identified a group of 13 significantly altered miRNAs, including miR-31, miR-96, miR-133b, miR-135b, miR-145, and miR-183 (Bandres et al., 2006). Downregulation of the miR-143/145 cluster has been repeatedly reported in colorectal cancer (Ibrahim et al., 2011; Chivukula et al., 2014), allowing further CRC therapeutic investigations. A first cancer-targeted miRNA drug- MRX34, a liposome- based miR-34 mimic, entered Phase I clinical trials in patients with advanced hepatocellular carcinoma in 2013 (Ling et al., 2013). The immediately observed and promising advantage of using microRNA approaches is based on the ability to concurrently target multiple effectors of pathways involved in cell differentiation, proliferation and survival, as opposed to targeting a single enzyme or transporter of the arginine metabolic network (which involve over 10 key enzymes and 2 critical transporters). However, there was little direct evidence that miRNAs affect the occurrence and development of CRC by regulating arginine metabolism. Since arginine metabolism is related to rectal cancer, searching the miRNAs targeted arginine metabolism-related enzyme may be new sights for the diagnosis and treatment of CRC.

**TABLE 2:** Circulating miRNAs changes associated with colorectal cancer.

**Study**	**Sample size**		**Source**	**Method of analysis**	**miRNA identified**	**Normalizer**	**Observations**
	**Patients controls**						
Ng et al. (2009)	120	90	Plasma	Microarray profiling and Validation by qPCR	miR-17-3p, miR-92, miR-95, miR-135b, miR-222, etc.	RNU6B	First study to evaluate circulating miRNA in CRC
Huang et al. (2010)	120	79	Plasma	qPCR on specific miRNAs	miR-29a, miR-92a	miR-16	Non-invasive biomarkers for early detection of CRC
Pu et al. (2010)	103	37	Plasma	qPCR on specific miRNAs	miR-221	N/A	86% sensitivity and 41% specificity in CRC
Liu et al. (2013)	200	80	Serum	qPCR on specific miRNAs	miR-21 and miR-92a	miR-16	Both miRNAs have potential value for early detection
Kanaan et al. (2012)	30	30	Plasma	Microarray profiling and Validation by qPCR	miR-21	U6	miR-21 differentiated CRC with 90% specificity and sensitivity
Zhang et al. (2013)	78	86	Plasma	qPCR on specific miRNAs	miR-18 and miR-200c	RNU6B	84.6% sensitivity and 75.6% specificity in CRC
Luo et al. (2013)	130	244	Plasma	Microarray profiling and Validation by qPCR	miR-18a, miR-20a, miR-21, miR-29a, miR-92a, miR-106b, miR-133a, miR-143, miR-145	miR-16	Potential use in a multi-marker blood based test for early detection of CRC
Toiyama et al. (2013)	198	65	Serum	qPCR on specific miRNAs	miR-21	cel-miR-39	92% sensitivity and 81% specificity in CRC
Wang et al. (2014)	113	89	Serum	qPCR on specific miRNAs	miR-21, miR-31, miR-92a, miR-181b, miR-203, let-7g	miR-16	Non-invasive biomarkers for early detection of CRC
Zanutto et al. (2014)	29	29	Plasma	qPCR on specific miRNAs	miR-21 and miR-378	miR-16	miR-378 discriminates CRC from healthy individuals
Du et al. (2014)	49	49	Plasma	qPCR on specific miRNAs	miR-21 and miR-92a	cel-miR-39	miR-21 had a higher diagnostic efficiency than miR-92a
Ogata-Kawata et al. (2014)	88	11	Serum (Exosome)	Microarray profiling and Validation by qPCR	miR-21, let-7a, miR-23a, miR- 150, miR-223, miR-1229, miR-1246	miR-451	First study to serum exosomal miRNAs in CRC
Basati et al. (2014)	40	40	Serum	qPCR on specific miRNAs	miR-21	RNU6B	77% sensitivity and 78% specificity in CRC
Lv et al. (2015)	146	60	Serum	qPCR on specific miRNAs	miR-155	N/A	Upregulated in CRC
Chen et al. (2015)	100	79	Plasma	qPCR on specific miRNAs	miR-20a and miR-106a	miR-16	miR-20a: 46% sensitivity and 73% specificity; miR-106a: 74 and 44%, respectively
Fang et al. (2015)	111	130	Plasma	qPCR on specific miRNAs	miR-24, miR-320a, miR-423-5p	cel-miR-39	Sensitivity: miR-24: 78%; miR-320a: 91%; miR-423: 89%

Long non-coding RNAs (lncRNAs) are the second most commonly studied ncRNAs in colorectal cancer, with increasing evidence of their implications in CRC specific gene expression and miRNAs. LncRNAs can act as miRNAs sponges and affect translation efficacy (Guttman and Rinn, 2012). Aberrant lncRNAs may have a functional role in the pathogenesis of colorectal cancer and clinical implications, such as HOTARI and MALAT1 (Gupta et al., 2010; Okugawa et al., 2015). Detecting interaction networks and causal relationships between the dysregulation of miRNAs/lncRNAs and hyperactivity of arginine metabolism will be offer insights into novel strategies for secondary prevention and treatment of colorectal cancer.

## Specific Targeting of Arginine Metabolism in Colorectal Cancer Treatment

Based on previously mentioned, hyperactive arginine metabolism play important roles in CRC development and development. Therefore, it is hopeful to inhibit this pathway for therapeutic purposes. For instance, drugs for ODC inhibition and SSAT induction have been used for cancer therapy. Nevertheless, the long-term efficacy needs further study.

The use of arginine deaminase (ADI) and arginine deprivation to inhibit tumor growth has made important progress, and many clinical trials are in progress, including in liver cancer, sarcoma and lymphoma (Phillips et al., 2013). However, it has to be emphasized that for various reasons this strategy cannot be very promising for CRC. *In vitro* experiments It have been shown that the intracellular arginine synthesis enzyme ASS is defective in many cancer, including renal cell carcinoma, HCC and so on, so that their growth is dependent on external arginine supplementation (Cheng et al., 2007; Kim et al., 2009; Delage et al., 2010) or intracellular synthesis with ASS and ASL. Thus, arginine deprivation (potentially also combined with ASL down- regulation) is only a viable option for cancers with defects in these enzymes.

Nevertheless, there is nevertheless a positive outlook in arginine-targeting in CRC. L-arginine is an important material for protein synthesis for human, and play important roles for all kind of cells (Rodriguez et al., 2007; Norian et al., 2009; Wu et al., 2009; Morris, 2010). Tumor-infiltrating immune cells cannot effectively uptake L-arginine in the tumor tissues (Rodriguez et al., 2007; Norian et al., 2009), thus, significantly increased L-arginine and L-Citrulline concentrations were found in CRC tissues (Mao et al., 2010). This indicated that L-arginine bioavailability is higher in the CRC tissue. In addition, ASS and ASL38 are high expression in colorectal cancer cells. Thus, arginine deprivation was rather expected to decrease the effectiveness of tumor-infiltrating cells, therefore, its function is limited on cancer cells. However, clinical data confirmed that L-arginine successfully improved cancer patient immunity, thus demonstrated a benefit of L-arginine as a supplement to the treatment of colorectal cancer (Ma et al., 2007). As such, we supposed that inhibiting arginine uptake by specifically targeting arginine transporters may be better for CRC than a systemic arginine deprivation. Studies on specific interventions in related regulatory mechanisms of L-arginine transport pathways and the innate compensation of each arginine transporter in CRC cells are ongoing in our team.

## Conclusion

Colorectal cancer is one of the common malignant tumors, but the curative effect of metastatic colorectal cancer is poor. With the deepening of research, more and more evidence shows that arginine metabolism is closely related to the occurrence and development of colorectal cancer. As polyamines are the most important metabolite of arginine, metabonomics analysis may be used in the diagnosis and screening of colorectal cancer in the future. In addition, arginine deprivation may be a viable option for cancer treatment, although further clinical trials are needed to confirm it. Because blocking arginine transporter can inhibit the uptake of arginine and inhibit the growth of tumor, blocking arginine transporter may be another potential anticancer strategy. Finally, more studies are needed to fully elucidate the regulatory role of arginine metabolism cycle in colorectal cancer.

miRNAs have been established as critical plays in colorectal cancer pathogenesis, early detection and prognosis. The advantage of using microRNA-based therapeutic is based on its ability to concurrently target multiple effectors of pathways involved in cancer cell differentiation, proliferation and survival, and arginine metabolism pathways. Therefore, applying one or two miRNAs cocktail targeting more than two enzymes or arginine transporters, such as targeting CAT-1 and ASS, should be expected more efficient in the treatment of colorectal cancer.

## Author Contributions

TD and JH wrote the manuscript. TD collected the data. JH reviewed and revised the manuscript. Both authors contributed to the article and approved the submitted version.

## Conflict of Interest

The authors declare that the research was conducted in the absence of any commercial or financial relationships that could be construed as a potential conflict of interest.
